# Recent Advances in Immunomodulatory Therapy in Sepsis: A Comprehensive Review

**DOI:** 10.7759/cureus.57309

**Published:** 2024-03-31

**Authors:** Abhishek Jain, Amol Singam, V N K Srinivas Mudiganti

**Affiliations:** 1 Critical Care Medicine, Jawaharlal Nehru Medical College, Datta Meghe Institute of Higher Education and Research, Wardha, IND

**Keywords:** clinical trials, treatment, immune dysregulation, inflammation, immunomodulatory therapy, sepsis

## Abstract

Sepsis remains a critical healthcare challenge, characterized by dysregulated immune responses to infection, leading to organ dysfunction and high mortality rates. Traditional treatment strategies often fail to address the underlying immune dysregulation, necessitating exploring novel therapeutic approaches. Immunomodulatory therapy holds promise in sepsis management by restoring immune balance and mitigating excessive inflammation. This comprehensive review examines the pathophysiology of sepsis, current challenges in treatment, and recent advancements in immunomodulatory agents, including biologics, immunotherapy, and cellular therapies. Clinical trial outcomes, safety profiles, and future research and clinical practice implications are discussed. While immunomodulatory therapies show considerable potential in improving sepsis outcomes, their successful implementation requires further research, collaboration, and integration into standard clinical protocols.

## Introduction and background

Sepsis is a life-threatening condition that arises when the body's response to infection causes widespread inflammation, leading to organ dysfunction and failure. It is characterized by a dysregulated immune response to infection, resulting in a cascade of inflammatory mediators and tissue damage [1]. Immunomodulatory therapy plays a crucial role in sepsis management by targeting the dysregulated immune response. By modulating the immune system, these therapies aim to restore immune balance, mitigate excessive inflammation, and prevent organ dysfunction, thereby improving patient outcomes [2].

Despite advances in medical care, sepsis remains a significant global health burden with high morbidity and mortality rates. Current treatment strategies primarily focus on antibiotics and supportive care, but they often fail to address the underlying immune dysregulation, leading to suboptimal outcomes. Additionally, the heterogeneity of sepsis presentation and the lack of reliable biomarkers pose challenges for early diagnosis and targeted therapy [3]. This review aims to provide a comprehensive overview of newer advancements in immunomodulatory therapy for sepsis. It will explore the pathophysiology of sepsis, discuss traditional treatment approaches, and examine recent developments in immunomodulatory agents. Furthermore, the review will evaluate the efficacy and safety of these novel therapies, identify challenges in their implementation, and discuss future directions for research and clinical practice.

## Review

Pathophysiology of sepsis

Immune Response in Sepsis

The sepsis pathophysiology entails an intricate interplay of immune responses and physiological abnormalities incited by infections. Sepsis is characterized by severe organ dysfunction resulting from a dysregulated host response to infection, with a renewed emphasis on immune pathophysiology [1]. Within sepsis, the body's reaction to infection becomes dysregulated, prompting a systemic release of cytokines, mediators, and molecules that activate coagulation and complement cascades, ultimately precipitating multi-organ dysfunction [1]. This dysregulation encompasses the upregulation of both pro- and anti-inflammatory pathways, which may culminate in tissue damage and immunosuppression during the later stages of the disease, rendering patients susceptible to secondary infections [1]. The pathogenesis of septic shock, an extreme manifestation of sepsis characterized by a profound drop in blood pressure, involves an inflammatory stimulus triggering the production of pro-inflammatory mediators such as tumor necrosis factor (TNF) and interleukin (IL)-1. These mediators elicit various effects, such as neutrophil-endothelial cell adhesion, clotting activation, and microthrombi formation, thereby contributing to organ dysfunction and hypoperfusion [4]. Initially, septic shock may manifest as warm shock with arterial dilation and increased cardiac output. Yet, it can progress to hypoperfusion, characterized by decreased cardiac output and blood pressure, leading to hallmark features of shock [4].

Dysregulation of Inflammatory Response

The dysregulation of the inflammatory response is pivotal in comprehending sepsis. Within the framework of sepsis 3, the term "systemic inflammatory response" has been supplanted by "dysregulated host response," underlining the intricate nature of the host's immune reaction to infection [5]. This dysregulation entails a multifaceted process encompassing inflammation, the neuroendocrine response, coagulation, and metabolic responses, all intricately intertwined in the pathophysiology of sepsis [5]. The inflammatory response in sepsis assumes a central role, spanning hyperinflammation in the early phase, defective inflammatory resolution, and persistent inflammation in the chronic phase of the condition [5]. Grasping the equilibrium between proinflammatory and anti-inflammatory processes, alongside their interactions with other host responses, such as neuroendocrine and coagulation systems, is critical in assessing the correlation between infection and organ dysfunction in sepsis [5].

The Role of Immunomodulation in Restoring Immune Balance

Immunomodulation is pivotal in reinstating immune equilibrium by orchestrating and fine-tuning the immune response. It entails the delicate balance of immune function to address instances where the immune response is either deficient or hyperactive. Through the modulation of the immune system, immunomodulatory therapies aspire to mitigate the clinical trajectory of diseases and reinstate homeostasis [6]. This process encompasses targeting various components of the immune system to devise interventions capable of effectively regulating immune responses [6]. Immunomodulation holds particular significance in conditions where chronic inflammation or aberrant immune responses contribute to the progression of diseases, such as autoimmune diseases and infections [6]. Using immunomodulation, the immune response can be externally tempered to forestall disease exacerbation and enhance patient outcomes by restoring the intricate balance of the immune system [6].

Traditional approaches to sepsis treatment

Antibiotics and Supportive Care

Antibiotics play a pivotal role in treating sepsis, aiming to target the underlying infection and impede its progression. The selection of antibiotics is guided by the presumed source of infection and the imperative for broad-spectrum coverage until specific pathogens are discerned via culture results. Empirical antibiotic therapy, typically comprising agents such as nafcillin, oxacillin, vancomycin, cephalosporin, daptomycin, or linezolid, is commonly initiated until more tailored, narrow-spectrum agents can be employed based on culture findings [7]. Timely administration of antibiotics is imperative, as studies have correlated early administration with improved survival outcomes in septic patients [8]. Furthermore, the duration of antibiotic therapy typically spans approximately two weeks, albeit this can fluctuate depending on the source, location, and severity of the infection [7].

Supportive care constitutes another crucial facet of sepsis treatment, concentrating on sustaining organ perfusion and furnishing respiratory support when necessary. Patients afflicted with sepsis are typically hospitalized, with admission to the intensive care unit (ICU) contingent upon the severity of the condition and degree of organ dysfunction [9]. The Surviving Sepsis Campaign underscores the significance of early and aggressive medical intervention for patients suspected of harboring sepsis, emphasizing the necessity for timely interventions to ameliorate patient outcomes [10]. In essence, a blend of appropriate antibiotics and supportive care is imperative in the comprehensive management of sepsis, endeavoring to address both the infection and the systemic inflammatory response to optimize patient care and outcomes.

Challenges and Limitations of Conventional Therapies

Conventional therapies for sepsis encounter notable challenges and constraints, necessitating innovative approaches to managing this intricate condition. A primary challenge lies in the broad and heterogeneous nature of sepsis, wherein patients with varied infections and organ dysfunctions often receive a generalized diagnosis of severe sepsis. This broad classification can impede the efficacy of treatments, as interventions may not be tailored to specific subsets of patients who could derive the most benefit from targeted therapies [11]. Furthermore, the frequent failure of clinical trials in sepsis treatment underscores the difficulty in pinpointing therapeutic targets capable of consistently enhancing survival rates in septic patients [11,12]. The limitations of conventional therapies are compounded by the excessive reliance on supportive measures such as systemic perfusion maintenance and infection eradication, which may not effectively address the underlying immune dysregulation propelling sepsis pathophysiology [11]. These challenges underscore the urgent imperative for innovative strategies that transcend traditional approaches to augment outcomes for sepsis patients.

Immunomodulatory therapy in sepsis: overview

Historical Perspective

The historical perspective on sepsis underscores the significant burden associated with this condition, with an estimated 700,000 cases occurring annually in North America and mortality rates ranging between 30% and 50% [13]. Extensive research has delved into the mechanisms underlying sepsis, utilizing experimental rodent models and human studies. These investigations have elucidated the excessive systemic production of reactive oxygen and nitrogen species, hyperinflammatory states, and immunosuppression, all of which compromise survival in sepsis patients [13]. Notably, publications in the American Journal of Pathology have played a pivotal role in disseminating research findings that explore the mechanisms of experimental sepsis, with a particular focus on elucidating the roles of factors such as peroxisome proliferator-activated receptors (PPARs), high levels of adhesion molecules, and receptors on immune cells and endothelial cells [13]. Moreover, the recognition of the long-term consequences of sepsis, including physical and cognitive impairments post-recovery, underscores the imperative for comprehensive treatment strategies that address both the acute and lingering effects of the condition [14]. The historical perspective also highlights the challenges encountered in clinical trials targeting proinflammatory mediators and receptors, underscoring the intricacy of the inflammatory and immune responses in sepsis and the necessity for judicious approaches in treatment design [15]. Additionally, potential alternative strategies such as immunostimulants like IL-15 and IL-7 are proposed to counteract the immunosuppressive state induced by sepsis, although further clinical trials are warranted to validate these approaches [16].

Types of Immunomodulatory Agents Used in Sepsis

**Figure 1:**
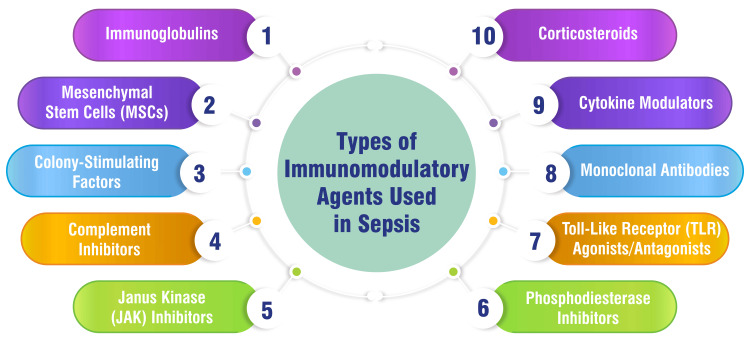
Types of immunomodulatory agents used in sepsis The image is created by the corresponding author.

The spectrum of immunomodulatory agents utilized in sepsis encompasses diverse treatments designed to modulate the immune response to improve patient outcomes. Traditional immunomodulation therapies such as corticosteroids, specific cytokine modifiers, complement or coagulation regulators, and growth factors have long been employed to manage sepsis [17]. However, recent advancements in research have led to the exploration of nonconventional immunomodulatory approaches to tackle the complexities of sepsis. These novel approaches include experimental drugs such as necrosulfonamide, which inhibits the oligomerization of gasdermin D (GSDMD), a molecule implicated in inflammatory responses [18]. Moreover, personalized immunotherapy has emerged as a promising strategy in sepsis treatment, focusing on tailoring interventions to individual patients based on their unique immune profiles [19]. This personalized approach aims to optimize treatment responses and minimize potential harm by targeting specific immune pathways that vary among patients. The evolving landscape of immunomodulatory therapies in sepsis reflects a shift toward precision medicine and individualized care, promising improved outcomes in managing this critical condition. Figure 1 shows the types of immunomodulatory agents used in sepsis.

Mechanisms of Action

The mechanisms underlying sepsis-induced immunosuppression and the actions of immunomodulatory therapies are intricate and multifaceted. Sepsis instigates an immune response characterized by a hyper-inflammatory phase succeeded by an immunosuppressive phase, prompting therapies directed at host immunity to enhance patient survival [20]. Several mechanisms contribute to sepsis-induced immunosuppression, including apoptosis of immune cells, heightened regulatory T cell activity, upregulation of programmed cell death 1 on CD4+ T cells, and cellular exhaustion [20]. Immunomodulatory molecules such as IL-7, IL-15, and anti-programmed cell death 1 have been identified as potential therapeutic targets to counteract immunosuppression in sepsis [20]. Furthermore, strategies involving immunostimulatory therapies such as interferon‐gamma (IFN-γ), granulocyte macrophage colony-stimulating factor (GM-CSF)), granulocyte colony-stimulating factor (G-CSF), IL-7, and IL-15 aim to invigorate the innate and adaptive immune systems to restore immunity in septic patients [20,21]. These approaches target various facets of the immune response to sepsis, underscoring the complexity inherent in immunomodulation for managing this critical condition.

Recent advances in immunomodulatory therapy

Biologics Targeting Specific Immune Pathways

Biologics represent a sophisticated class of drugs designed to precisely target specific immune system components, offering a more refined approach to treating various conditions. These medications obstruct particular aspects of the immune system responsible for autoimmune diseases and inflammation, facilitating more targeted and efficacious treatments [22]. Diverse classes of biologics target specific inflammatory pathways, encompassing TNF inhibitors, IL-1 inhibitors, IL-6 inhibitors, IL-17 inhibitors, IL-12 inhibitors, and IL-23 inhibitors, as well as B-cell and T-cell inhibitors, each tailored to address distinct immune components implicated in conditions such as rheumatoid arthritis, psoriasis, Crohn's disease, among others [22]. By impeding the actions of cytokines and modulating immune cell functions, biologics furnish a personalized therapeutic strategy that mitigates broad immune responses, consequently reducing the risk of adverse effects commonly associated with traditional immunotherapies [22].

Immunotherapy Approaches Checkpoint Inhibitors and Cytokine Therapies

Recent advancements in immunotherapy approaches, particularly focusing on checkpoint inhibitors and cytokine therapies, have revolutionized cancer treatment. Checkpoint inhibitors, including programmed cell death protein 1 (PD-1), programmed cell death-ligand 1 (PD-L1), and cytotoxic T-lymphocyte-associated protein 4 (CTLA-4) inhibitors, have remarkably succeeded in clinical trials across various cancer types. Drugs such as pembrolizumab, cemiplimab, nivolumab, atezolizumab, avelumab, and durvalumab have exhibited promising results in treating malignancies such as non-small cell lung cancer, melanoma, kidney cancer, and more [23]. These inhibitors obstruct receptors that cancer cells exploit to evade T-cell attacks, thereby amplifying T-cell responses against tumors [23]. Additionally, combination therapies involving checkpoint inhibitors, such as ipilimumab and nivolumab, have demonstrated substantial survival benefits in patients with metastatic melanoma [24]. Concurrently, cytokine therapies that modulate the immune response are being investigated as potential treatments. The synergy between immune checkpoint blockade and conventional therapies like surgery, chemotherapy, and radiation is actively explored to potentiate antitumor T-cell responses and enhance treatment outcomes [25]. Furthermore, developing personalized combination therapies and neoantigen-based cancer vaccines represents a cutting-edge approach to cancer treatment, aiming to achieve durable, safe, and target-specific chemotherapy [23]. These advancements underscore the dynamic nature of immunotherapy, underscoring the significance of tailored treatments and combination strategies to optimize patient outcomes in cancer therapy.

Cellular Therapies: Mesenchymal Stem Cells (MSCs) and Immune Cell Therapy

MSCs have emerged as a focal point in cellular therapies, garnering substantial attention owing to their remarkable immunomodulatory properties and therapeutic potential. These versatile cells can regulate immune responses, rendering them invaluable in treating various immune-mediated conditions [26,27]. MSC therapy is a promising avenue for addressing inflammatory diseases, providing a novel approach to mitigating immune dysregulation and inflammation [28]. Furthermore, the immunomodulatory capabilities of MSCs have spurred their investigation into cancer therapy, underscoring their potential in cell-based strategies for managing diverse diseases, including cancer [29]. The robust immunomodulatory prowess of MSCs positions them as a promising resource for precision clinical applications, accentuating their systemic safety profile and favorable biodistribution for tailored therapeutic interventions [30].

Nanotechnology-Based Interventions

**Figure 2:**
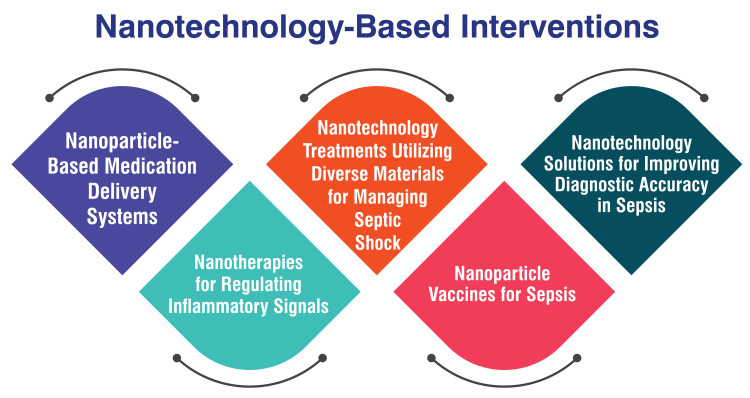
Nanotechnology-based interventions The image is created by the corresponding author.

Nanotechnology-based interventions have emerged as promising avenues in managing sepsis, with recent research showcasing significant potential. These interventions harness nanomaterials for both the diagnosis and treatment of sepsis. Nanoparticle-based delivery of antibiotics has yielded encouraging results in combatting drug resistance in sepsis studies, offering a promising avenue for improved treatment outcomes [31]. Furthermore, nanotherapies engineered to regulate inflammatory signals and intercept pathogen-associated molecular patterns (PAMPs) and damage-associated molecular patterns (DAMPs) hold immense promise for treating sepsis by precisely targeting the underlying inflammatory responses [32]. Additionally, nanotechnology-based treatments employing diverse materials have effectively managed septic shock and enhanced survival rates in preclinical models [33]. These advancements underscore the burgeoning role of nanotechnology in furnishing more precise and efficient diagnostic and therapeutic solutions for sepsis, thus offering hope for enhanced clinical outcomes in the future. Figure 2 shows nanotechnology-based interventions.

Efficacy and safety of novel immunomodulatory therapies

Clinical Trial Outcomes

The outcomes of clinical trials in sepsis research have been subject to intense scrutiny and analysis in recent years. Recent studies have shed light on the challenges encountered in designing effective trials to improve outcomes for patients with sepsis. One critical aspect pertains to selecting appropriate outcome measures for phase III trials, emphasizing endpoints that hold clinical significance for patients, such as survival duration and time to progression [34]. Despite concerted efforts to enhance trial designs, including the validation of new monitoring devices and the evaluation of innovative therapies like immunomodulation, the results of recent large-scale sepsis trials have consistently shown a primary endpoint of short-term all-cause mortality, with limited improvements in survival rates observed [35]. The complexity of sepsis as a syndrome characterized by the involvement of multiple pathways and systems necessitates a multifaceted treatment approach that either targets various pathways with multiple interventions or focuses on upstream nodes to modulate the immune response [35] effectively. Furthermore, the heterogeneity observed in the sepsis patient population presents challenges in trial design, with mortality rates influenced by factors extending beyond traditional scoring systems, such as the Sequential Organ Failure Assessment (SOFA) score, underscoring the importance of gaining a comprehensive understanding of patient characteristics and responses to interventions [36]. These findings underscore the ongoing need for evolution and refinement in clinical trial methodologies to effectively address the complexities inherent in sepsis and ultimately improve patient outcomes.

Adverse Effects and Safety Concerns

The sources provide valuable insights into immunomodulatory therapies' adverse effects and safety concerns. While immunotherapy represents a rapidly evolving field with significant benefits, it also poses challenges due to its potential adverse effects. These therapies, encompassing monoclonal antibodies and immunomodulators, can potentially induce systemic toxicities affecting various organ systems, including cardiovascular, dermatologic, endocrine, gastrointestinal, neurologic, and pulmonary systems [37,38]. Commonly reported adverse effects such as injection-site reactions, fatigue, flulike symptoms, and infusion-associated reactions underscore the importance of diligent monitoring and patient education to manage these side effects effectively [39,40]. Moreover, the risk of immune reactions, including acute anaphylaxis and cytokine release syndrome, underscores the necessity for cautious administration and vigilant monitoring of monoclonal antibodies to mitigate the occurrence of severe adverse events [38]. Overall, comprehending and addressing these adverse effects are pivotal in ensuring immunomodulatory therapies' safe and effective utilization in clinical practice.

Patient Selection and Personalized Approaches

Patient selection and personalized approaches in cancer immunotherapy represent pivotal aspects of modern oncology. The field of immuno-oncology underscores the importance of tailoring treatments to individual patients based on their unique characteristics to optimize therapeutic outcomes. Personalized immuno-oncology entails the integration of various technologies and the selection of the most appropriate ones for each patient, encompassing immune checkpoint inhibitors, monoclonal antibodies, immunogene therapy, and vaccines [41]. Biomarkers play a significant role in predicting responses to immunotherapy, with molecular diagnostics and sequencing guiding treatment decisions in immuno-oncology [41]. Genomic profiling of tumor mutational burden enhances the predictive utility of immune checkpoint inhibitors such as PD-1/PD-L1 drugs, underscoring the significance of personalized approaches in cancer treatment [41]. Furthermore, patient-specific cancer immunotherapies are gaining traction by identifying individual mutations, neoantigens, and biomarkers facilitated by advances in genomics and proteomics [42]. These advancements promise to expand the responder patient population by tailoring treatments based on patient-specific factors. Engineering approaches, including biomaterial design, delivery strategies, and nanotechnology solutions, are harnessed to develop individualized cancer treatments, such as nanoparticle vaccines and cell therapies tailored to each patient [42]. The future of personalized cancer immunotherapy lies in the synergistic combination of different strategies, including neoantigen peptide delivery, gene therapy employing nanoparticles, personalized cellular therapies, and image-guided theragnostic approaches, to achieve enhanced anti-tumor effects and improve patient outcomes [42].

Challenges and future directions

Heterogeneity of Sepsis Patients

The heterogeneity of sepsis patients represents a complex and multifaceted challenge that impedes the development of precise and effective treatments. Research has elucidated that sepsis is a heterogeneous syndrome characterized by a diverse array of clinical and biological features, thereby complicating the advancement of therapeutic approaches beyond current standards [43]. This heterogeneity stems from various factors, including host-related elements such as age, biological sex, comorbidities, genetics, infection etiology, dysregulated host responses, and multiple organ dysfunctions [44]. Studies have demonstrated that sepsis patients exhibit disparate disease manifestations, progression trajectories, and responses to treatment owing to this heterogeneity, underscoring the imperative for personalized and tailored approaches to sepsis management [45].

Biomarkers for Patient Stratification

Biomarkers play a pivotal role in patient stratification for sepsis management, facilitating the identification of high-risk subgroups and guiding personalized treatment approaches. Several biomarkers have exhibited promise in enhancing diagnostic accuracy and prognostic enrichment in clinical trials. Notably, procalcitonin has emerged as a valuable biomarker for early sepsis prediction, aiding in identifying patients with infection and organ dysfunction [46]. Combining procalcitonin with clinical scores such as the quick Sepsis-related Organ Failure Assessment (qSOFA) offers a straightforward yet effective screening tool for early sepsis detection in the emergency department [46]. Moreover, mid-regional pro-adrenomedullin (MR-proADM) has been assessed as a predictive biomarker to refine mortality stratification in sepsis patients, showcasing its potential to enhance patient outcomes through improved risk assessment [47]. Additionally, metabolomic studies have delved into metabolic phenotyping as a means of patient stratification in sepsis, aiming to unravel the condition's heterogeneity and identify distinct metabolic profiles associated with organ dysfunction, disease severity, and treatment response [48].

Optimization of Treatment Protocols

Implementing enhanced protocol compliance measures involves regular education sessions on sepsis, utilizing technology-based resources for training, and assessing outcomes post-intervention to gauge improvements within the healthcare system [49]. By providing ongoing education and training sessions, healthcare providers can stay updated on the latest sepsis protocols and guidelines, ensuring adherence to best practices in sepsis management. Utilizing technology-based resources such as online modules and interactive training tools can further enhance the effectiveness of education initiatives, allowing for more flexible and accessible learning opportunities. Assessing outcomes, post-intervention enables healthcare organizations to measure the impact of protocol compliance efforts and identify areas for further improvement, ultimately leading to better patient outcomes.

Patient-specific digital precision diagnostics are crucial in sepsis management by utilizing next-generation sequencing (NGS) for precise pathogen detection [50]. This approach allows for targeted antimicrobial treatment strategies based on the specific pathogens identified in individual patients, optimizing treatment efficacy and reducing the risk of antimicrobial resistance. By leveraging advanced diagnostic technologies like NGS, healthcare providers can make more informed decisions regarding antimicrobial therapy, improving outcomes for sepsis patients.

Sepsis performance improvement programs focus on optimizing compliance with sepsis care protocols, enhancing diagnostic strategies, and improving the overall care of sepsis patients throughout hospitalization [51]. These programs aim to drive better outcomes by standardizing sepsis care practices, implementing evidence-based guidelines, and promoting interdisciplinary collaboration among healthcare teams. By continuously monitoring and evaluating sepsis care processes, performance improvement programs can identify areas for enhancement and implement targeted interventions to improve patient care and outcomes.

New recommendations advocate for retiring outdated measures such as the Centers for Medicare and Medicaid Services (CMS) Severe Sepsis/Septic Shock Management (SEP-1), instead of focusing on sepsis mortality rates [52]. By refining diagnostic strategies, optimizing care throughout hospitalization, and enhancing rehabilitation services for sepsis survivors, healthcare organizations can improve patient outcomes and reduce mortality rates associated with sepsis. These recommendations emphasize the importance of adopting a comprehensive approach to sepsis management that addresses all aspects of patient care, from early detection and diagnosis to post-discharge support and rehabilitation.

The Surviving Sepsis Campaign Guidelines 2021 highlight the importance of involving patients and their families in care discussions, coordinating early follow-up post-discharge, and implementing performance improvement programs for sepsis screening and treatment in hospitals and health systems [53]. By engaging patients and their families in care planning and decision-making, healthcare providers can improve patient satisfaction and outcomes. Coordinating early follow-up post-discharge ensures continuity of care and allows for timely intervention if complications arise. Implementing performance improvement programs for sepsis screening and treatment helps standardize care practices and promote adherence to evidence-based guidelines, ultimately leading to better outcomes for sepsis patients.

## Conclusions

In conclusion, this review has underscored the pivotal role of immunomodulatory therapy in managing sepsis, a condition marked by a dysregulated immune response to infection leading to organ dysfunction and failure. Through an exploration of sepsis's definition, pathophysiology, and current treatment challenges, the review has illuminated the pressing need for novel therapeutic approaches. Recent advancements in immunomodulatory agents, including biologics, immunotherapy, and cellular therapies, offer promising avenues for intervention. These therapies aim to restore immune balance, mitigate excessive inflammation, and improve patient outcomes. Future research endeavors should focus on elucidating the underlying mechanisms of immune dysregulation in sepsis, identifying biomarkers for patient stratification, and conducting large-scale clinical trials to evaluate the efficacy and safety of novel therapies. In clinical practice, integrating these therapies into sepsis management protocols, particularly for patients with severe or refractory disease, holds significant potential to reduce mortality rates associated with sepsis. Ultimately, immunomodulatory therapies can revolutionize sepsis management and enhance patient outcomes globally with continued research and collaboration.
